# MEPIRAPIM-derived synthetic cannabinoids inhibit T-type calcium channels with divergent effects on seizures in rodent models of epilepsy

**DOI:** 10.3389/fphys.2023.1086243

**Published:** 2023-04-04

**Authors:** Thomas Harman, Michael Udoh, Dan L. McElroy, Lyndsey L. Anderson, Richard C. Kevin, Samuel D. Banister, Adam Ametovski, Jack Markham, Chris Bladen, Peter T. Doohan, Quentin Greba, Robert B. Laprairie, Terrance P. Snutch, Iain S. McGregor, John G. Howland, Jonathon C. Arnold

**Affiliations:** ^1^ The Lambert Initiative for Cannabinoid Therapeutics, Brain and Mind Centre, The University of Sydney, Sydney, NSW, Australia; ^2^ Discipline of Pharmacology, Sydney Pharmacy School, Faculty of Medicine and Health, The University of Sydney, Sydney, NSW, Australia; ^3^ Department of Anatomy, Physiology and Pharmacology, University of Saskatchewan, Saskatoon, SK, Canada; ^4^ School of Chemistry, Faculty of Science, The University of Sydney, Sydney, NSW, Australia; ^5^ Department of Biomedical Sciences, Macquarie University, Sydney, NSW, Australia; ^6^ College of Pharmacy and Nutrition, University of Saskatchewan, Saskatoon, SK, Canada; ^7^ Michael Smith Laboratories and Djavad Mowafaghian Centre for Brain Health, University of British Columbia, Vancouver, BC, Canada; ^8^ School of Psychology, Faculty of Science, The University of Sydney, Sydney, NSW, Australia

**Keywords:** cannabinoid, epilepsy, mepirapim, Dravet syndrome, GAERS, 6 Hz, Ca_v_3 channels

## Abstract

**Background:** T-type Ca^2+^ channels (Ca_v_3) represent emerging therapeutic targets for a range of neurological disorders, including epilepsy and pain. To aid the development and optimisation of new therapeutics, there is a need to identify novel chemical entities which act at these ion channels. A number of synthetic cannabinoid receptor agonists (SCRAs) have been found to exhibit activity at T-type channels, suggesting that cannabinoids may provide convenient chemical scaffolds on which to design novel Ca_v_3 inhibitors. However, activity at cannabinoid type 1 (CB_1_) receptors can be problematic because of central and peripheral toxicities associated with potent SCRAs. The putative SCRA MEPIRAPIM and its analogues were recently identified as Ca_v_3 inhibitors with only minimal activity at CB_1_ receptors, opening the possibility that this scaffold may be exploited to develop novel, selective Ca_v_3 inhibitors. Here we present the pharmacological characterisation of SB2193 and SB2193F, two novel Ca_v_3 inhibitors derived from MEPIRAPIM.

**Methods:** The potency of SB2193 and SB2193F was evaluated *in vitro* using a fluorometric Ca^2+^ flux assay and confirmed using whole-cell patch-clamp electrophysiology. *In silico* docking to the cryo-EM structure of Ca_v_3.1 was also performed to elucidate structural insights into T-type channel inhibition. Next, *in vivo* pharmacokinetic parameters in mouse brain and plasma were determined using liquid chromatography-mass spectroscopy. Finally, anticonvulsant activity was assayed in established genetic and electrically-induced rodent seizure models.

**Results:** Both MEPIRAPIM derivatives produced potent inhibition of Ca_v_3 channels and were brain penetrant, with SB2193 exhibiting a brain/plasma ratio of 2.7. SB2193 was further examined in mouse seizure models where it acutely protected against 6 Hz-induced seizures. However, SB2193 did not reduce spontaneous seizures in the *Scn1a* ^*+/−*^ mouse model of Dravet syndrome, nor absence seizures in the Genetic Absence Epilepsy Rat from Strasbourg (GAERS). Surprisingly, SB2193 appeared to increase the incidence and duration of spike-and-wave discharges in GAERS animals over a 4 h recording period.

**Conclusion:** These results show that MEPIRAPIM analogues provide novel chemical scaffolds to advance Ca_v_3 inhibitors against certain seizure types.

## 1 Introduction

T-type channels (Ca_v_3) are low-voltage-activated plasma membrane channels which selectively mediate calcium conductance in many types of electrically excitable cells (Zamponi et al., 2015). Mammalian genomes possess three genes which encode for distinct Ca_v_3 channel isoforms: *CACNA1G* (Ca_v_3.1), *CACNA1H* (Ca_v_3.2), and *CACNA1I* (Ca_v_3.3) (Catterall et al., 2005). Ca_v_3 channels activate at hyperpolarised membrane potentials, exhibit small unitary conductance, and rapid activation and inactivation kinetics (Perez-Reyes, 2003). These unique biophysical properties allow T-type channels to play a critical role in the regulation of neuronal excitability and to affect burst firing and oscillatory activity in neuronal circuits (Cain and Snutch, 2013). Ca_v_3-mediated slow wave oscillations in thalamocortical neurons underlie non-REM sleep rhythmogenesis (Crunelli et al., 2006). Thalamocortical oscillations are also implicated in the generation of spike-and-wave discharges (SWDs) which occur in absence epilepsy (Cain et al., 2018).

Aberrant Ca_v_3 activity is associated with a range of neurological disorders, including neuropathic pain and epilepsy (Cain and Snutch, 2013; Weiss and Zamponi, 2020). In preclinical models of neuropathic pain increased expression of T-type channels in dorsal root ganglia neurons and increased activity of Ca_v_3.2 channels in primary afferent fibres have been observed (Yue et al., 2013; Bourinet et al., 2016). Mutations in the Ca_v_3.1-encoding gene *CACNA1G* and Ca_v_3.2-encoding gene *CACNA1H* are associated with idiopathic generalised epilepsies in humans (Weiss and Zamponi, 2020). Genetic Absence Rats from Strasbourg (GAERS) possess a gain-of-function missense mutation in *Cacna1h* which underlies the propagation of absence seizures in these animals (Cain et al., 2018). In mice, genetic overexpression of *Cacna1g* produces a pure absence epilepsy phenotype (Ernst et al., 2009). *Cacna1g* has also been identified as a genetic modifier of epilepsy in the *Scn1a* ^*+/−*^ mouse model of Dravet syndrome (Calhoun et al., 2017). Genetic knockdown of *Cacna1g* improves survival and reduces the frequency of spontaneous seizures in *Scn1a* ^*+/−*^ mice (Calhoun et al., 2017). In contrast, mutations in Ca_v_3.3 have not been associated with epilepsy or pain to date, although Ca_v_3.3 protein expression is upregulated in animal models of trigeminal neuropathic pain (Montera et al., 2021). Interestingly, Ca_v_3.3 null mice exhibit increased susceptibility to drug-induced SWDs (Lee et al., 2014).

Consequently, T-type channels have emerged as pharmacological targets of interest for the treatment of pain and seizure disorders (Weiss and Zamponi, 2019). Indeed, inhibition of Ca_v_3 channels appears to contribute to the therapeutic effect of the anticonvulsant drugs ethosuximide and zonisamide (Gomora et al., 2001; Matar et al., 2009). Recently, several potent pan Ca_v_3 channel blockers have been developed which exhibit analgesic and anticonvulsant effects in preclinical models. Two of these compounds, Z944 and ACT-709478, have advanced to phase II clinical trials for the treatment of pain and generalised epilepsy, respectively (Nam, 2018). In preclinical studies, Z944 exhibited analgesic effects in the Freund’s adjuvant model of inflammatory pain, as well as anticonvulsant effects in GAERS and both the amygdala kindling and kainic acid models of temporal lobe epilepsy (Tringham et al., 2012; Casillas-Espinosa et al., 2015; Casillas-Espinosa et al., 2019; Harding et al., 2021). ACT-709478 is efficacious in preclinical models of absence epilepsy and generalised convulsive seizures (Bezençon et al., 2017).

The identification of novel chemical scaffolds with actions at specific T-type channel isoforms may further aid in the development and optimisation of new therapeutics. Several endogenous, plant-derived, and synthetic cannabinoids inhibit T-type channels *in vitro* suggesting that cannabinoid ligands provide promising scaffolds with which to design novel Ca_v_3 inhibitors (Chemin et al., 2001; Mirlohi et al., 2021; Mirlohi et al., 2022). MEPIRAPIM, a putative synthetic cannabinoid receptor agonist (SCRA), and its analogues were recently described as inhibitors of Ca_v_3 subtypes (Kevin et al., 2022). An issue with most SCRAs is that they have potent effects on cannabinoid CB_1_ receptors leading to central and peripheral toxicities (Courts et al., 2016; Alipour et al., 2019). However, we recently made the surprising discovery that MEPIRAPIM and several analogues display negligible activity at CB_1_ receptors, thus opening the possibility that this chemical scaffold could be exploited to develop novel, selective Ca_v_3 inhibitors (Kevin et al., 2022).

**FIGURE 1:**
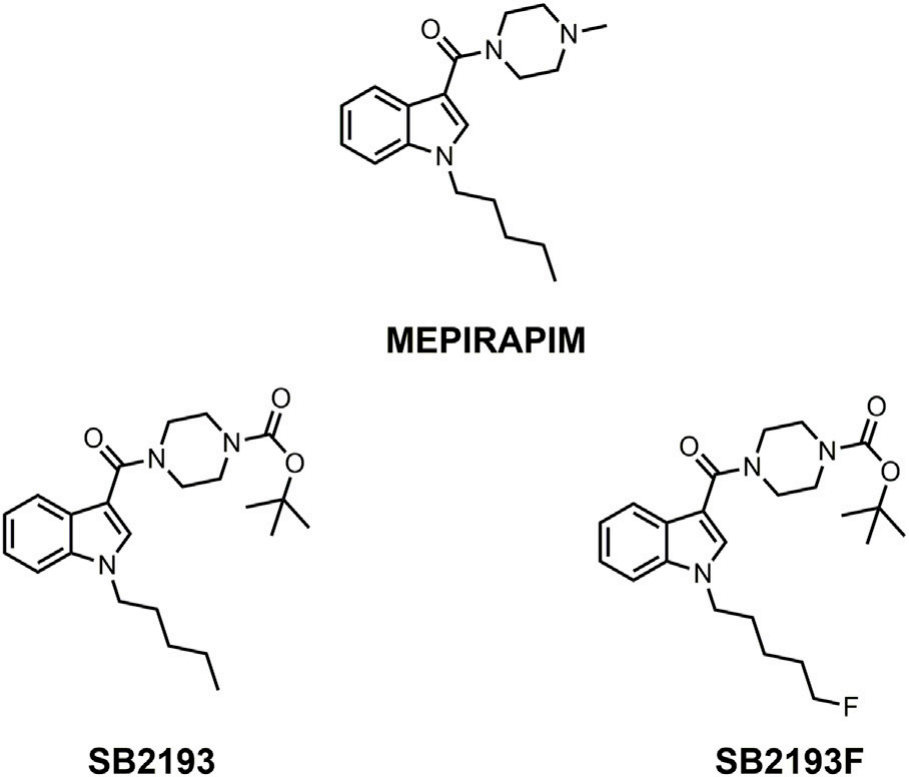
Chemical structures of MEPIRAPIM and its analogues.

Here we report the pharmacological characterisation of two novel T-type channel inhibitors derived from MEPIRAPIM, designated SB2193 and SB2193F (Figure 1). These compounds feature a Boc group attached to the piperazine ring of the MEPIRAPIM scaffold, with the addition of a fluorine to the terminal hydrocarbon chain to generate SB2193F. We aimed to initially determine the inhibitory potency of these compounds at the three Ca_v_3 subtypes and then to determine their brain and plasma pharmacokinetic profiles. These data were then used to guide subsequent *in vivo* experiments to establish potential anti-seizure effects of these compounds in rodent models of seizures and epilepsy.

## 2 Materials and methods

### 2.1 Chemicals

SB2193 and SB2193F were synthesised by Dr Samuel Banister at the University of Sydney, Australia as previously described (Kevin et al., 2022). NNC 55-0396 was obtained from Tocris (Bristol, United Kingdom). Diazepam, tetracaine, and valproic acid were purchased from Sigma-Aldrich (St Louis, United States). For sub-chronic administration, compounds were formulated in irradiated rat and mouse standard chow (Specialty Feeds, Glen Forest, Australia).

### 2.2 Cell culture

HEK293 FlpIn T-Rex cells (Invitrogen, CA, United States) stably expressing human Ca_v_3.1, Ca_v_3.2, or Ca_v_3.3 were maintained in Dulbecco’s Modified Eagle Medium (DMEM) supplemented with 10% foetal bovine serum (FBS), 1% penicillin-streptomycin (P/S) at 37°C, and 5% CO_2_ as previously described (Bladen et al., 2021; Udoh et al., 2022). Hygromycin (80 μg/mL) and blasticidin (15 μg/mL) were used as selection antibiotics and cells were passaged at 80% confluency. For experiments, cells were resuspended in Leibovitz’s L-15 media (containing 1% FBS, 1% P/S, 15 mM glucose, 2 μg/mL tetracycline), plated in black, clear-bottomed poly-D-lysine-coated 96-well plates (90 μL) (Corning, NY, United States), and incubated overnight in ambient air at 37°C. All compounds tested in cellular assays were prepared as stock solutions in DMSO and final DMSO concentration was 0.1%.

### 2.3 Calcium flux assay

Intracellular calcium flux was measured using a fluorometric imaging plate-reader (FLIPR) Calcium 5 Assay Kit and FlexStation 3 Microplate Reader (Molecular Devices, San Jose, United States). Calcium 5 dye (90 μL) prepared in low potassium HBSS (Thermo Fisher Scientific) containing (in mM) 145 NaCl, 22 HEPES, 0.338 Na_2_HPO_4_, 4.17 NaHCO_3_, 0.441 KH_2_PO_4_, 0.407 MgSO_4_, 0.493 MgCl_2_, 1.26 CaCl_2_, 5.56 glucose, and 250 probenecid with pH 7.4 was applied to each well and incubated for 1 h at 37°C, ambient air prior to testing.

Fluorescence readings were taken at 2 s intervals (*λ*_excitation_ = 485 nm, *λ*_emission_ = 525 nm). Following 2 min baseline reading, 20 μL of SB2193, SB2193F, or NNC 55-0396 (10 nM—10 μM) was added to cells and read for 5 min, then CaCl_2_ (10 mM) was added and read for 3 min. Baseline fluorescence averaged over the 20 s interval preceding CaCl_2_ addition was subtracted and normalized to the vehicle response. Changes in fluorescence were measured in relative fluorescence units (RFU). All compounds were assayed in ≥ five independent experiments (*n* = 5–8) performed in duplicate. Order of compound addition and location on the plate were varied between replicates. Data was fit to a four-parameter non-linear dose-response curve using GraphPad Prism 8.2.0 software (La Jolla, United States) to determine IC_50_ values.

### 2.4 Whole-cell patch-clamp electrophysiology

All whole-cell voltage-clamp recordings from HEK293 FlpIn T-Rex cells stably transfected with human Ca_v_3.1 were performed at room temperature. At least 24 h prior to experiments, cells were detached from flasks using trypsin/EDTA and plated into 10 cm sterile tissue culture dishes containing 10 mL of supplemented DMEM and 10–15 glass coverslips (12 mm diameter, ProScitech, QLD, Australia). Culture dishes were then kept overnight in same conditions as flasks to allow cells to adhere to coverslips. They were then transferred to a 30°C/5% CO_2_ incubator to inhibit cell proliferation until ready to be used for electrophysiology experiments. At this stage, channel expression was induced with tetracycline (2 μg/mL, Sigma-Aldrich) 8–24 h prior to recording cells.

External recording solutions contained (in mM): 114 CsCl, 5 BaCl_2_, 1 MgCl_2_, 10 HEPES, 10 glucose, adjusted to pH 7.4 with CsOH. The internal patch pipette solution contained (in mM): 126.5 CsMeSO_4_, 2 MgCl_2_, 11 EGTA, 10 HEPES adjusted to pH 7.3 with CsOH. Internal solution was supplemented with 0.6 mM GTP and 2 mM ATP and mixed thoroughly just before use. Liquid junction potentials for the above solutions were calculated before experiments using pClamp 10 software and corrected during experiments. Compounds were prepared daily from 30 mM DMSO stocks and diluted into an external solution just before use. Compounds were then applied rapidly and locally to the cells using a custom-built gravity-driven micro-perfusion system (Feng et al., 2003). Initial vehicle experiments were performed to ensure that 0.1% DMSO did not affect current amplitudes or channel kinetics (data not shown), and all subsequent experiments contained 0.1% DMSO in control external solutions. Currents were elicited from a holding potential of −100 mV and were measured by conventional whole-cell patch clamp techniques using an Axopatch 200B amplifier in combination with Clampex 9.2 software (Molecular Devices, Sunnyvale, CA). After establishing whole cell configuration, cellular capacitance was minimised using the amplifier’s built-in analogue compensation. Series resistance was kept to <10 MΩ and was compensated to at least 85% in all experiments. All data were digitised at 10 kHz with a Digidata 1,320 interface (Molecular Devices) and filtered at 1 kHz (8-pole Bessel filter). Raw and online leak-subtracted data were both collected simultaneously, P/N4 leak subtraction was performed using opposite polarity and after the protocol sweep.

For tonic inhibition of T-type currents, membrane potential was stepped from −100 mV to −30 mV for 200 m and then allowed to recover for 12 s (1 sweep). A minimum of 10 sweeps were collected under vehicle control external perfusion to allow for control peak current to equilibrate. The drug SB2193 was then continuously perfused, and sweeps were recorded until no further inhibition was seen (minimum of 3 sweeps with the same amplitude).

### 2.5 Molecular modelling

The cryo-EM structure of Ca_v_3.1 (PDB: 6KZP) was retrieved from RCSB PDB (rcsb.org) (Berman et al., 2003; Zhao et al., 2019). The structure was prepared with Maestro’s Protein Preparation Wizard as follows (Madhavi Sastry et al., 2013). The phospholipid, cholesterol, and glucopyranose molecules were removed, as well as the Ca^2+^ atoms, leaving only the ion channel and its cognate ligand, Z944. The preparation process consisted of assigning bond orders, adding hydrogens, generation of disulphide bonds, generation of missing side chains using Prime, and generation of het states using Epik at pH 7.5 ± 1.0 (Jacobson et al., 2004; Shelley et al., 2007). The hydrogen bonding network was optimized, the pKa values of the protein were predicted using PROPKA and target pH value was set at 7.5 (Olsson et al., 2011). Lastly, the protein structure was minimized using the OPLS4 force field where RMSD of the atom displacement for terminating the minimization was set as 0.3 Å (Lu et al., 2021).

Ligands SB2193 and SB2193F were prepared using LigPrep to generate energy minimized 3D structures (Schrödinger Release 2022–3 Ligprep, 2021). OPLS4 force field was used for minimization. Epik was used to generate all possible ionized states at pH 7.5 ± 1.0. A receptor grid was generated using Glide, with a Van der Waals radius scaling factor of 1.0 and a partial charge cut-off at 0.25 (Friesner et al., 2006). The binding site was defined by the centroid of the cognate ligand. The Van der Waals scaling factor for the ligands was set to 1.0 with a partial charge cut-off at 0.15. The precision was set to Extra Precision (XP) with flexible ligand sampling. Nitrogen inversions and ring conformations were sampled.

### 2.6 Animals

All mouse seizure and pharmacokinetic experiments were approved by the University of Sydney Animal Ethics Committee (protocols 2019/1687, 2020/1736, and 2018/1395) and conducted in accordance with the Australian Code of Practice for the Care and Use of Animals for Scientific Purposes (2013). All mice were maintained on a 12 h light/dark cycle with lights on at 0700 and access to food and water *ad libitum*.

*Scn1a* ^*+/−*^ mice purchased from The Jackson Laboratory (stock 37107-JAX; Bar Harbour, United States) were maintained as a congenic line on a 129S6/SvEvTac background (129. *Scn1a* ^+/−^). F1 mice were generated by crossing 129. *Scn1a* ^+/−^ mice with wild-type C57BL/6J mice (Jackson Laboratory stock no. 000664) and group housed. *Scn1a* genotype was determined as previously described (Miller et al., 2014).

Swiss outbred mice used in 6 Hz seizure experiments were purchased from Australian Resources Centre (stock ARC(S); Canning Vale, Australia) and single housed for 1 week prior to experimentation to prevent fighting.

GAERS experiments were conducted in accordance with the standards of the Canadian Council on Animal Care and approved by the University of Saskatchewan Animal Research Ethics Board (protocol 20130118). GAERS were initially sourced from the University of Melbourne to establish a Canadian GAERS colony in 2010 (Powell et al., 2014) and weanlings were provided by the Snutch Laboratory at the University of British Columbia to the University of Saskatchewan in 2013 (snutchlab.msl.ubc.ca) (Marks et al., 2016; Marks et al., 2019). GAERS were maintained on a 12 h light/dark cycle with lights on at 0700 and access to food and water *ad libitum* except during EEG recordings.

### 2.7 Pharmacokinetic studies

SB2193 and SB2193F were prepared in ethanol-Tween 80%–0.9% saline (1:1:18 ratio) and valproic acid in 0.9% saline. All doses were completely soluble in this vehicle except for the 100 mg/kg SB2193 dose, which was a suspension.

Male and female wildtype F1 mice aged 21–28 days postnatal received a single i. p. injection of 10 mg/kg SB2193 or SB2193F. At selected timepoints (15–240 min), mice were anesthetised with isoflurane, blood was collected *via* cardiac puncture and whole brains were harvested. Brains were snap frozen on dry ice, and blood plasma was isolated *via* centrifugation (4,000 *g* for 10 min, 4^○^C). Brain and plasma samples were stored at −80°C until assayed.

### 2.8 Analytical methods

Plasma samples (50 μL) were spiked with an internal standard (diazepam) and vortexed with 1 M NaOH (50 μL) and methyl *tert*-butyl ether (1 mL) to achieve protein precipitation. The organic layer was isolated by centrifugation (4,000 *g* for 3 min) before being frozen for 60 min at −80°C, evaporated to dryness using N_2,_ and reconstituted in 30% acetonitrile and 0.1% formic acid in water for analysis. Brain hemispheres were homogenised in a 10x w/v 1:1 methanol-water solution using a Biospec Tissue-Tearor (Bartlesville, United States) and centrifuged at 20,000 *g* for 30 min at 4°C. Supernatant (100 μL) was collected and prepared following the same method as plasma.

SB2193 and SB2193F concentrations in brain and plasma samples were assessed by high performance liquid chromatography-tandem mass spectrometry (HPLC-MS/MS) using a Shimadzu Nexera liquid chromatograph coupled with a Shimadzu 8,030 triple quadrupole mass spectrometer (Kyoto, Japan). Samples were run through a Zorbax C18 HPLC Column equipped with a Zorbax Eclipse XDB-C8 guard column (2.1 × 50 mm, 3.5 μm particle size; Agilent, Santa Clara, United States) with an injection volume of 10 μL using a gradient elution. Mobile phases of acetonitrile and 0.1% formic acid in water were delivered at a flow rate of 0.6 mL/min over 4.16 min, with a stepwise adjustment of mobile phase concentration gradient over time to optimise ion separation.

Mass transitions and collision energy were optimised for each compound in multiple reaction monitoring mode using electrospray ionised positive mode, as displayed in Supplementary Table S1. Quantification was achieved by comparing experimental samples to plasma and brain standards prepared with known concentrations of SB2193 or SB2193F. Samples were quantified against a 7-point standard curve (*R* ^2^ > 0.98 for both analytes).

Concentrations of SB2193 or SB2193F in plasma and brain at each timepoint were averaged and concentration-time curves were generated using GraphPad Prism. Pharmacokinetic parameters were calculated using non-compartmental analysis. The linear-log trapezoidal method was used to calculate drug exposure (area under the curve, AUC) using equations previously described (Hawkins et al., 2017).

### 2.9 Spontaneous seizures and survival monitoring in *Scn1a* ^*+/−*^ mice

On P18, male and female *Scn1a* ^*+/−*^ mice were weaned and randomly assigned to treatment groups (untreated or SB2193) with *n* = 17–19 per group. For spontaneous seizure and survival experiments, SB2193 was administered sub-chronically *via* supplementation of the compound into chow to avoid stress associated with repeated i.p. injections. Chow was formulated at 5,000 mg/kg SB2193 in chow to provide target brain concentrations of 0.73 μM, with the daily oral dose based on the assumption that each mouse consumes approximately 3 g chow/day. Bodyweight of mice was monitored during the administration period to ensure consumption of chow. Animals in both treatment groups gained weight at an equivalent rate over the chow supplementation period (Supplementary Figure S1).

Continuous video recordings from 00:00 P23 to 24:00 P24 were made using a Samsung SCB-5003 days/night camera equipped with an infrared lens (Seoul, South Korea). The number of spontaneous generalised tonic-clonic seizures (GTCS) occurring over the 48 h recording period was quantified offline by a blinded observer. Statistical comparison was performed in GraphPad Prism using a Mann-Whitney *U* test as data were non-parametric. Treatment was continued to P30 to monitor survival of mice. *Scn1a* ^*+/−*^ mice have a high premature mortality rate, with approximately 50% of animals surviving to P30 (Miller et al., 2014). We have observed substantial variability in the mortality rate across experimental cohorts which is difficult to explain and likely due to stochastic factors (Anderson et al., 2020; Anderson et al., 2021; Anderson et al., 2022). Statistical comparison of survival across groups was performed in GraphPad Prism using Mantel-Cox log rank test.

### 2.10 6 Hz seizures in mice

6 Hz seizures were induced in Swiss male mice (9–10 weeks old) using an Ugo Basile electroconvulsive therapy unit (Model 57,800; Gemonio, Italy). Mice were pre-treated with vehicle, SB2193 (10 mg/kg, 30 mg/kg, 100 mg/kg) or valproic acid (300 mg/kg) *via* i.p. injection 15 min before seizure induction based on the time-to-peak brain concentration determined in pharmacokinetic studies. Valproic acid was administered at 300 mg/kg based on previous studies that showed this dose suppressed seizures in the 6 Hz assay (Benson et al., 2020; Koneval et al., 2020). A drop of 0.5% tetracaine was applied to both corneas immediately following the i.p. injection to provide local anaesthesia. Immediately prior to the electrical stimulation, saline was applied to each cornea to ensure electrical conductivity. Corneal electroshocks were administered at 44 mA, 6 Hz, 3 s shock duration, with a 0.2 m rectangular pulse width. Mice were shocked and scored by an observer blinded to treatment for the presence of twitching of the vibrissae, stun, myoclonus, rearing, forelimb clonus, and/or Straub tail. Animals were scored as protected if they displayed normal behaviour without signs of seizure in the 30 s period following shock. The ED_50_ for SB2193 was calculated using computer-assisted log-probit linear regression analysis (Litchfield and Wilcoxon, 1949). A χ^2^ test was performed to assess statistical significance.

### 2.11 GAERS experiments

GAERS display reliable spike and wave discharges (SWD) characteristic of childhood absence epilepsy (CAE) (Tringham et al., 2012; Marks et al., 2019; Roebuck et al., 2021; McElroy et al., 2022; Roebuck et al., 2022). Experiments were conducted in male (*n* = 11; two removed due to poor recording integrity) and female (*n* = 5) GAERS. Group sizes were determined based on previous experiments and availability of age, sex, and litter matched animals (Marks et al., 2019; McElroy et al., 2022; Roebuck et al., 2022). Animals were well handled and habituated to the recording protocol prior to testing. Baseline recordings were performed prior to sessions with drug treatment and SB2193 (10, 30, 100 mg/kg) or vehicle (5 mL/kg) was administered (i.p.) ∼15–20 min prior to recordings using a within-subjects design (5–7 days washout periods between treatments). SB2193 was freshly prepared in ethanol-Kolliphor (Sigma Aldrich)-saline (1:1:18 ratio).

### 2.12 EEG surgery and recordings

Construction and implantation of electrodes proceeded according to established protocols (Farrell et al., 2018; Marks et al., 2019; McElroy et al., 2022; Roebuck et al., 2022). Surgeries were performed under isoflurane anaesthesia with buprenorphine, anafen, and bupivicaine (topical) used as analgesics. Bipolar electrodes were chronically implanted bilaterally in somatosensory cortex (A/P 0.6  mm, M/L 5.5  mm, D/V −3.0  mm, relative to bregma) (Polack et al., 2007). Implants were secured with 3.2 mm stainless steel screws (one served as a ground) and dental cement. Animals were given a 1-week recovery period before habituation and testing.

Following recovery from surgery, animals were handled for 3–4 days and habituated to the EEG tether and hook-up process at least once before baseline recordings. Local field potentials (LFP) were acquired by tethered EEG (Grass Technologies), amplified 5,000x, and digitized at 100 Hz (Farrell et al., 2018; Marks et al., 2019; McElroy et al., 2022; Roebuck et al., 2022). Recordings (4 h) were conducted in two clear Plexiglas boxes (32 cm × 32 cm) and treatments were randomized such that each animal received each treatment. During recording sessions, animals were prevented from sleeping with gentle taps on the chamber door as necessary. Animals were perfused to confirm electrode placement following testing.

SWDs were analysed using a custom MATLAB (MathWorks) script developed by Dr. Stuart Cain and Jeff LeDue at the University of British Columbia. SWDs were assessed between 7 and 12 Hz and defined as bursts > 3x baseline amplitude >0.5 s as previously described (Powell et al., 2014; Farrell et al., 2018; Marks et al., 2019; Roebuck et al., 2021; McElroy et al., 2022; Roebuck et al., 2022). SWDs were analysed semi-automatically using Matlab and manually confirmed by a researcher blinded to treatment. A second researcher re-analysed a subset of files and interrater reliability was confirmed. Treatment data are normalized to initial non-treatment baseline data (e.g., % change) to account for SWD variability between subjects. Raw SWD data are also reported for comparison.

Time-binned data were analysed using a two-way analysis of variance (ANOVA) with factors of treatment (vehicle, 10, 30, 100 mg/kg SB2193) and time (1, 2, 3, 4 h post-treatment). Raw EEG data (e.g., not normalized to baseline) were then analysed using a repeated-measures one-way ANOVA. Post-hoc analyses were conducted with Tukey’s test or Dunnett’s multiple comparison test (compared to vehicle). Statistics were analysed in GraphPad Prism (version 9.0.1) and statistical significance was set at *p* ≤ 0.05. Post hoc testing was not performed unless *p* ≤ 0.05. All values are reported as mean ± SEM unless otherwise stated.

## 3 Results

### 3.1 MEPIRAPIM analogues are potent inhibitors of Ca_v_3 channels

The MEPIRAPIM analogues SB2193 and SB2193F strongly inhibit T-type channels at a single 10 μM concentration as shown using a FLIPR calcium flux assay and whole-cell patch clamp electrophysiology; however, a full concentration-response analysis had not been conducted to enable potency and subtype selectivity to be determined (Kevin et al., 2022). To this end, we investigated the concentration-dependent effects of these compounds across the different Ca_v_3 channel subtypes using a FLIPR calcium flux assay in Ca_v_3.1, Ca_v_3.2, and Ca_v_3.3 transfected HEK293 cells. The potent pan T-type channel inhibitor NNC 55-036 was used as a positive control.

**FIGURE 2:**
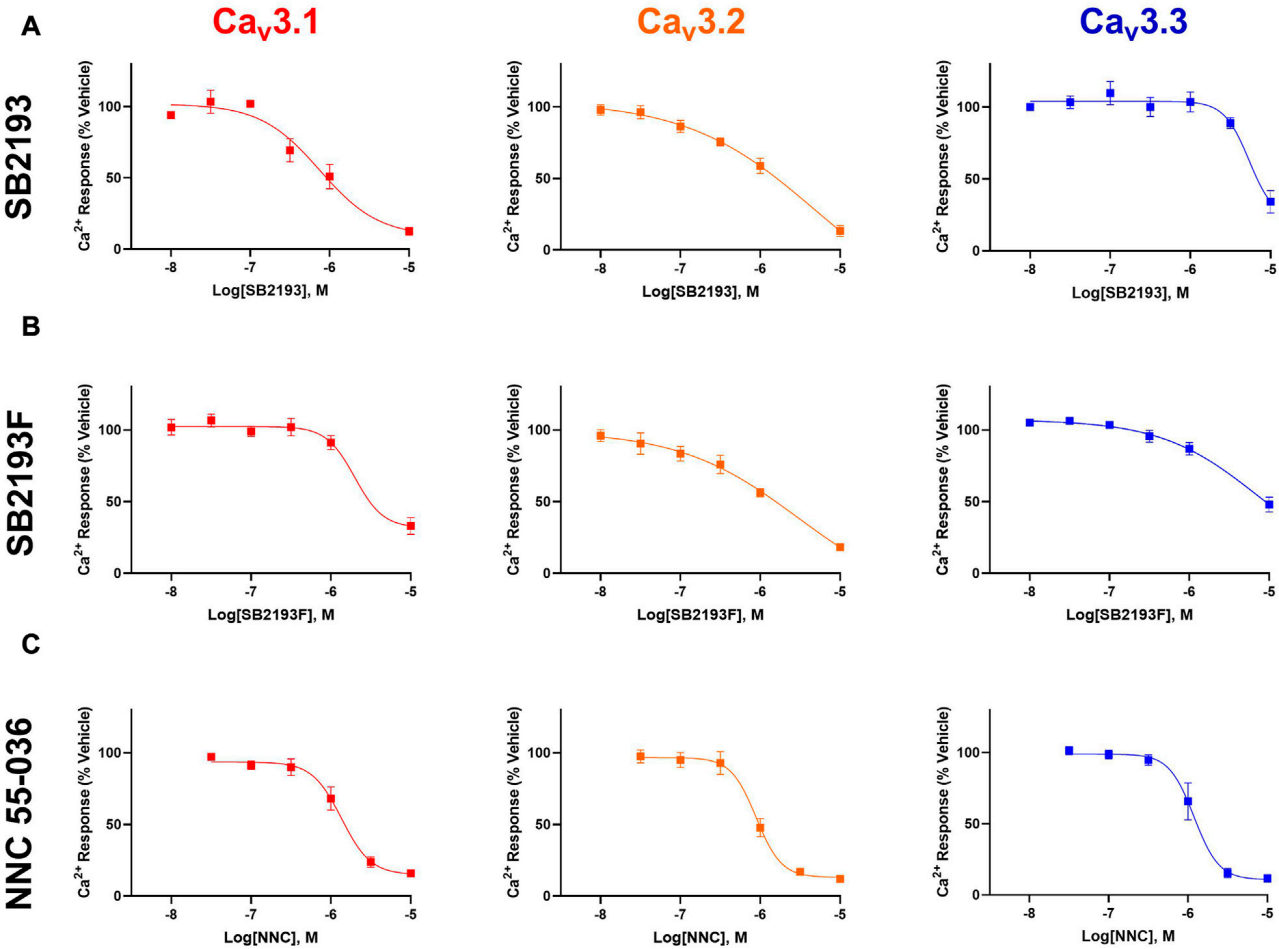
MEPIRAPIM analogues are potent inhibitors of Ca_v_3 channels. Concentration-response curves of SB2193 **(A)**, SB2193F **(B)**, and NNC 55-036 **(C)** at Ca_v_3 channel subtypes (*n* = 5–8). Calcium response was measured in the presence of varying concentrations of each compound as measured using a fluorometric imaging plate-reader (FLIPR) calcium flux assay and expressed as a percentage of response to vehicle. Data are expressed as mean ± SEM and curves represent fit to a four-parameter log function.

SB2193 most potently inhibited Ca_v_3.1 (IC_50_ = 750 nM), and less potently inhibited Ca_v_3.2 (IC_50_ = 5.9 μM) and Ca_v_3.3 (IC_50_ = 5.4 μM). SB2193F showed low micromolar potency across all three T-types, with IC_50_ values of 2.3 μM at Ca_v_3.1, 3 μM at Ca_v_3.2, and 8.6 μM at Ca_v_3.3. Both compounds displayed comparable potency to NNC 55-036 at Ca_v_3.1 but were less potent at Ca_v_3.2 and Ca_v_3.3 (Table 1; Figure 2).

**TABLE 1:** Half-maximal inhibitory concentration (IC_50_) values of inhibitors at Ca_v_3 channel subtypes.

	Ca_v_3.1		Ca_v_3.2		Ca_v_3.3	
	IC_50_ (μM)	pIC_50_	IC_50_ (μM)	pIC_50_	IC_50_ (μM)	pIC_50_
SB2193	0.75	6.1 ± 0.14	5.9	5.2 ± 1.4	5.4	5.3 ± 1.1
SB2193F	2.3	5.6 ± 0.93	3	5.5 ± 1.2	8.6	5.1 ± 1.9
NNC 55-036	1.3	5.9 ± 0.06	0.89	6 ± 0.05	1.2	5.9 ± 0.06

**FIGURE 3:**
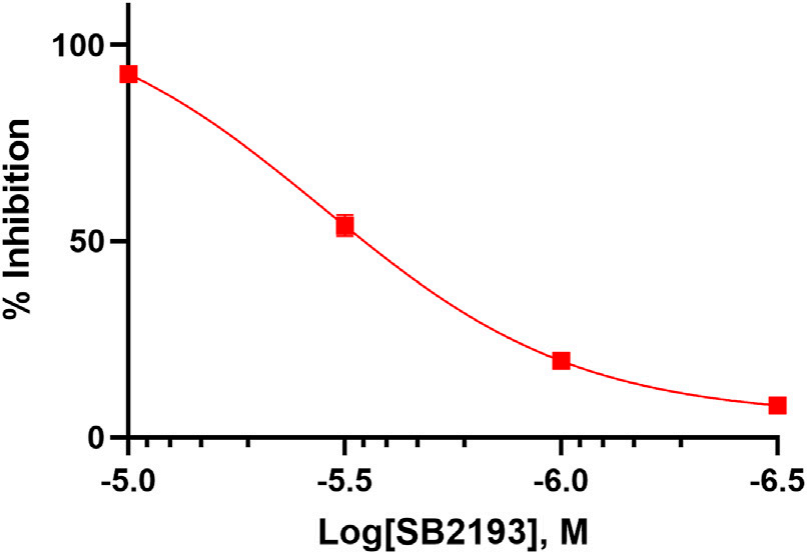
SB2193 inhibited Ca_v_3.1 in whole-cell patch-clamp electrophysiology experiments (*n* = 7–8). Data are expressed as mean ± SEM and curves represent fit to a four-parameter log function.

The inhibitory potency of SB2193 at Ca_v_3.1 was further validated by whole cell patch-clamp electrophysiology (Figure 3). SB2193 displayed inhibitory activity, although the potency of this compound in this assay (IC_50_ = 3.5 μM, pIC_50_ = 5.45 ± 0.07) was approximately 4-fold less than that determined by the FLIPR assay.

### 3.2 Molecular modelling

To provide further structural insight into the inhibition of Ca_v_3 channels by SB2193 and SB2193F, we performed docking for each of the ligands using a cryo-EM structure of Ca_v_3.1 (PDB ID: 6KZP) retrieved from the RCSB Protein Databank (Berman et al., 2003; Zhao et al., 2019).

**FIGURE 4:**
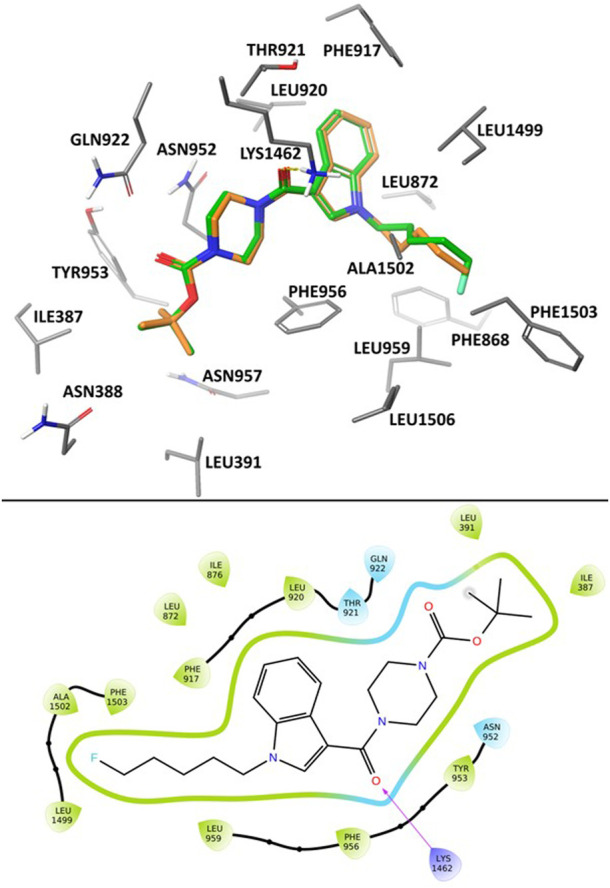
Calculated binding mode of SB2193 (orange) and SB2193F (green) at Ca_v_3.1 (PDB: 6KZP) (Zhao et al., 2019) using GlideXP Docking (Friesner et al., 2006) (top). 2D Ligand Interaction Diagram depicting the summarizing the interactions between SB2193F and the Ca_v_3.1 binding site (bottom).

In the poses calculated for the two ligands using Glide XP docking there was a high degree of uniformity observed in their binding position and protein-ligand interactions (Figure 4). Most notably, we observed hydrogen-bond coordination between the amide carbonyl oxygen and Lys1462. The polar piperazine and ester groups were stabilised *via* coordination with nearby residues Asn952, Gln922 and Thr921. Hydrophobic interactions between the pentyl and 5F-pentyl substituents of the compounds and hydrophobic binding site residues including Phe1503, Ala1502, Leu959, and Leu1499. The tert-butyl group is stabilised through interaction with Leu391 and Ile387. Additionally, coordination of the aromatic indole structure with the aromatic residues Phe956 and Phe917 are observed in calculated docking poses.

The association between inhibition and coordination to the residues described here is in agreement with previous observations of available Ca_v_3.1 crystal structures and their bound agonists (Zhao et al., 2019).

### 3.3 Pharmacokinetic parameters of MEPIRAPIM analogues

**FIGURE 5:**
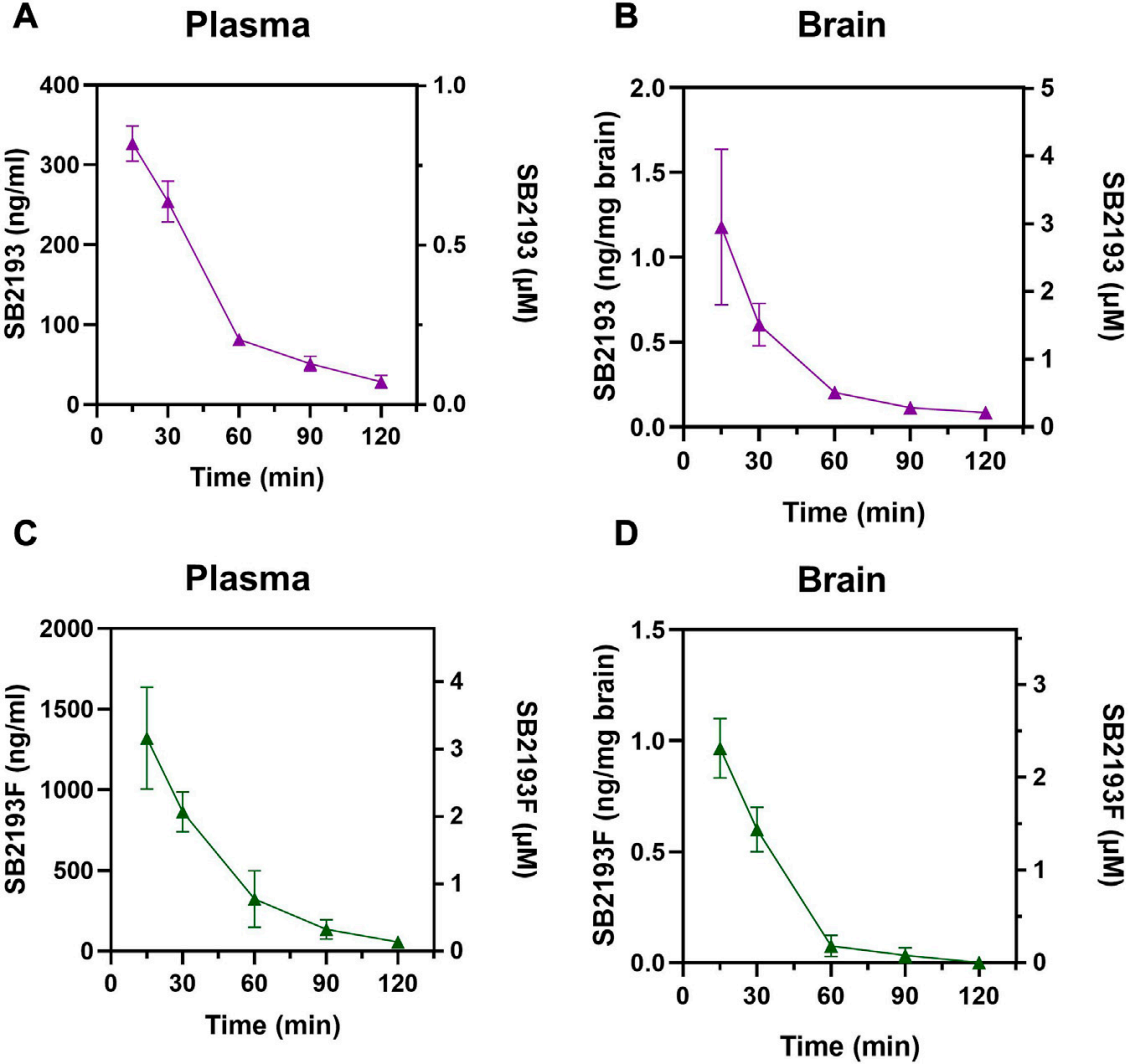
Pharmacokinetic analysis of MEPIRAPIM analogues in mouse plasma and brain samples. Concentration-time curves for SB2193 in mouse plasma **(A)** and brain **(B)** and SB2193F in mouse plasma **(C)** and brain **(D)** following 10 mg/kg i.p. injections. Data are expressed as mean ± SEM with *n* = 4 per timepoint.

We next determined the pharmacokinetic parameters of SB2193 and SB2193F in mouse brain and plasma following i.p. injection at 10 mg/kg. Data are shown in Table 2; Figure 5. Both SB2193 and SB2193F were rapidly absorbed, reaching peak plasma and brain concentrations within 15 min post-administration (Figure 5). Elimination was rapid with short brain and plasma half-lives for SB2193 (plasma = 29 min, brain = 28 min) and SB2193F (plasma = 23 min, brain = 14 min). Both compounds were brain penetrant and attained low micromolar concentrations, with brain-plasma ratios of 2.7 for SB2193 and 0.48 for SB2193F. Notably, although the maximal concentration (C_max_) of SB2193F in brain was nearly equivalent to the C_max_ in plasma, due to a substantially shorter half-life its total exposure was less. Given the superior brain penetrance and longer half-life of SB2193, we proceeded to *in vivo* studies with this compound only.

**TABLE 2:** Pharmacokinetics of SB2193 and SB2193F in mouse plasma and brain following single intraperitoneal injection (10 mg/kg).

	SB2193		SB2193F	
	Plasma	Brain	Plasma	Brain
C_max_ (*μ*g/mL)	0.3 ± 0.02	1.2 ± 0.4	1.3 ± 0.3	0.97 ± 0.1
C_max (_ *μ*M)	0.75 ± 0.05	3 ± 1	3.1 ± 0.7	2.3 ± 0.3
*t*_max_ (min)	≤15	≤15	≤15	≤15
*t*_1/2_ (min)	29	28	23	14
AUC (*μ*g min/mL)	13	35^a^	44	21^a^
brain-plasma ratio	2.7		0.48	

^a^ Brain concentration (ng/mL) converted from measured concentration (ng/mg brain) assuming density of 1 g/mL.

### 3.4 Examining the anticonvulsant effects of SB2193 in *Scn1a* ^*+/−*^ and 6 Hz mouse models of epilepsy

Given that transgenic reduction of Ca_v_3.1 expression reduced spontaneous seizure frequency is *Scn1a* ^*+/−*^ mice, we reasoned that administration of a T-type channel inhibitor may ameliorate seizure burden in this model (Calhoun et al., 2017). The effect of sub-chronic SB2193 treatment on spontaneous seizure frequency and survival in *Scn1a* ^*+/−*^ mice was assessed by administering 5,000 mg/kg SB2193 orally in chow from P18—P30. Administration of chow *ad libitum* to male and female mice (*n* = 10) from P18–23 achieved brain concentrations of 687 ± 227 ng/mg (1.7 ± 0.6 μM) which was within the target µM concentration range.

**FIGURE 6:**
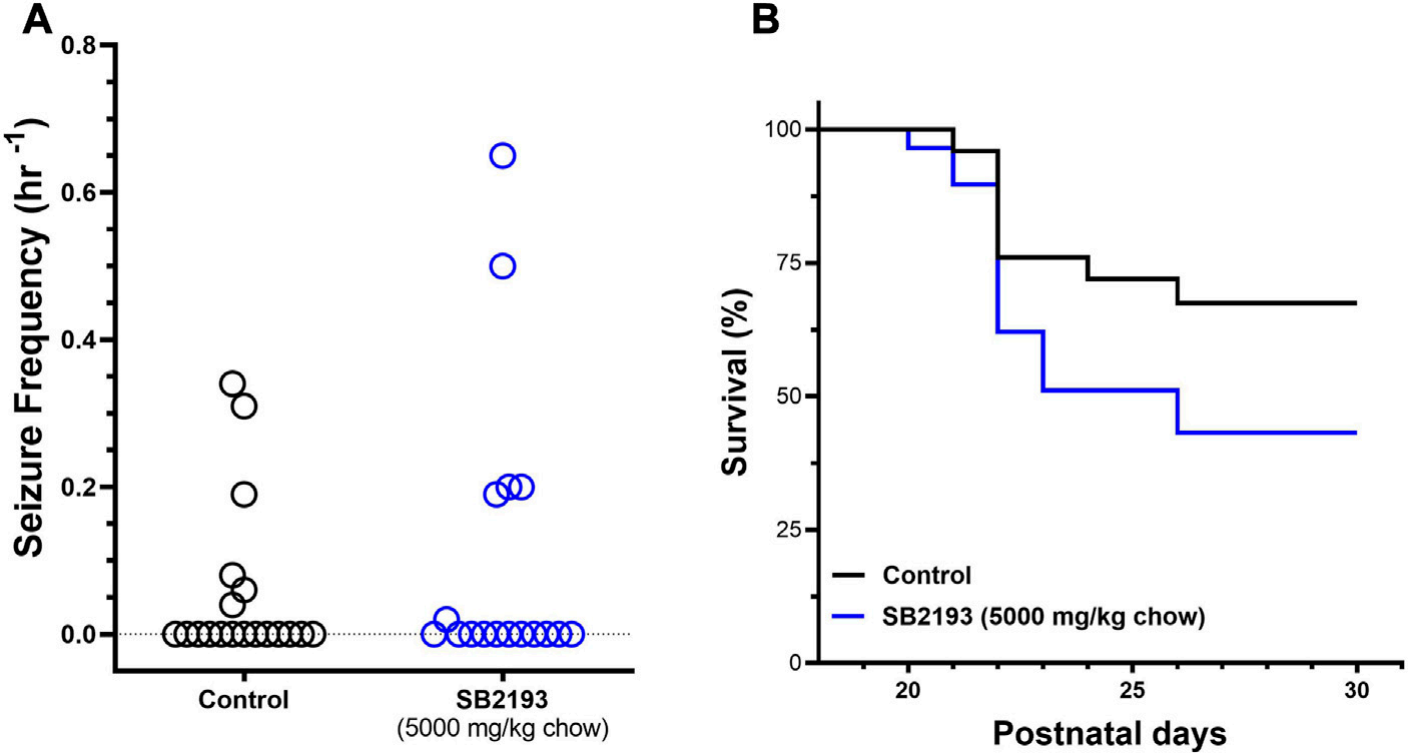
SB2193 treatment in *Scn1a* ^*+/−*^ mice. Spontaneous generalised tonic-clonic seizure (GTCS) frequency of individual untreated and SB2193-treated *Scn1a* ^*+/−*^ mice **(A)**. Treatment was administered orally through supplementation in chow (5,000 mg/kg chow) from postnatal day 18 and spontaneous GTCSs were quantified over 48 h from postnatal day 23–24. Sub-chronic SB2193 treatment had no effect on spontaneous seizure frequency (*p* = 0.67, Mann-Whitney *U* test, *n* = 17–19). Survival curves comparing untreated and SB2193-treated *Scn1a* ^*+/−*^ mice **(B)**. SB2193 treatment had no effect on survival of *Scn1a* ^*+/−*^ mice to P30 (*p* = 0.09, Mantel-Cox log rank).

SB2193 treatment did not decrease spontaneous seizure frequency over the 48 h recording window from P23-24 compared to control mice (Figure 6A). Further, SB2193 treatment had no effect on survival of mice to P30 (Figure 6B).

**FIGURE 7:**
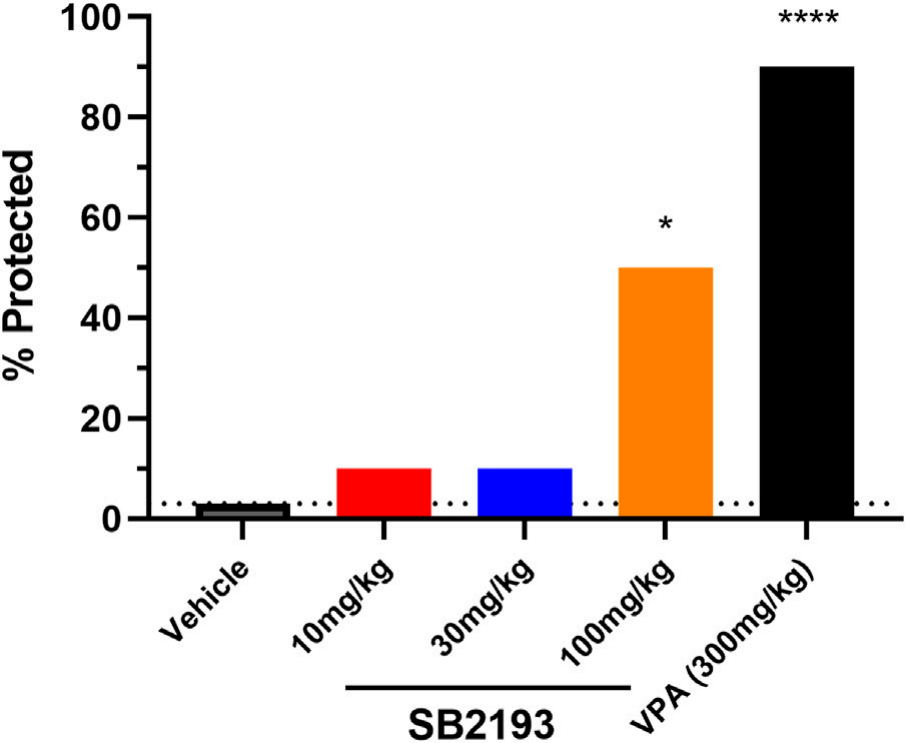
SB2193 treatment in Swiss mice administered 6 Hz corneal stimulation. Percentage of Swiss mice protected from seizure in the 6 Hz seizure model following acute treatment with SB2193 or valproic acid (*n* = 10 per group). Drugs were administered *via* intraperitoneal injection. SB2193 (100 mg/kg) increased the percentage of mice protected (χ^2^ = 6.33, *p* = 0.01) as did valproic acid (300 mg/kg) (χ^2^ = 15.54, *p* < 0.001) (*p < 0.05, ****p ≤ 0.0001).

We next assessed the effect of acute i.p. injection of SB2193 in the mouse 6 Hz model of psychomotor seizures (Figure 7). SB2193 (100 but not 10 or 30 mg/kg) significantly increased the percentage of mice protected from seizures compared to vehicle (χ^2^ = 6.33, *p* = 0.01). As a positive control for this model, valproic acid (300 mg/kg) provided significant protection (χ^2^ = 15.54, *p* < 0.001). Log-probit analysis was used to calculate the ED_50_ of SB2193, which was estimated as approximately 120 mg/kg.

### 3.5 SB2193 dose-dependently increases SWD incidence and duration in GAERS

**FIGURE 8:**
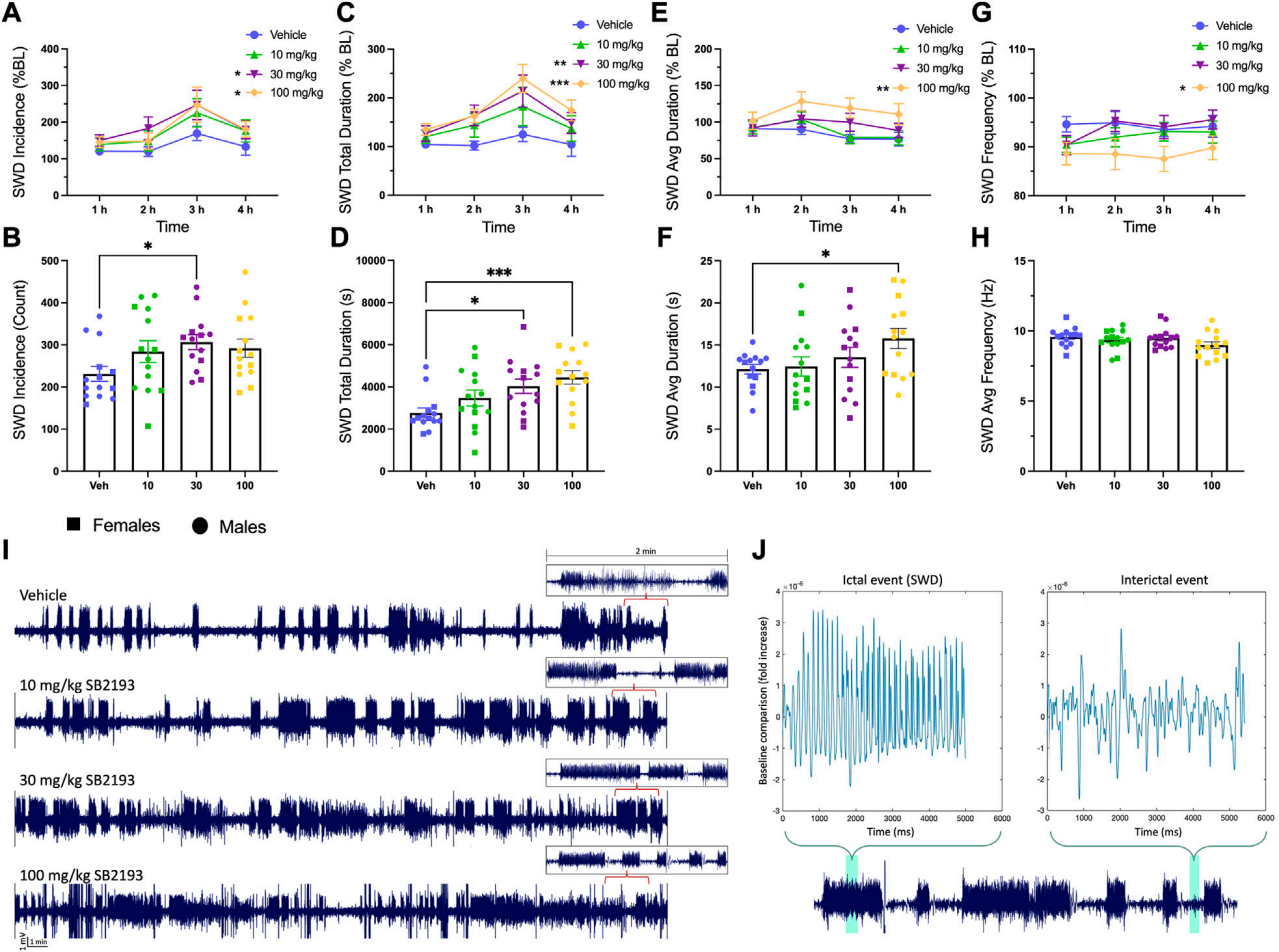
SB2193 increased spike and wave discharge (SWD) incidence and duration in GAERS (males: *n* = 9; females: *n* = 5). SB2193 treatments (Veh, 10, 30, 100 mg/kg) were administered (i.p.) ∼20 min prior to EEG recordings (4 h). SWDs are reported as the normalized percentage change from baseline **(A, C, E, G)** and raw data sampled over the 4 h recording window **(B, D, F, H)**. Representative EEG traces show 30 min samples from a single rat following the four treatments; insets depict zoomed-in 2 min traces **(I)**. SWD event durations were assessed semi-autonomously using Matlab (script = “EEG Seizure Analyzer”) and confirmed by an experimenter based on pre-established criteria (7–12 Hz; burst activity > 3x baseline for at least >0.5 m). **(J)** Panels show a Matlab-produced SWD event (top left) alongside an interictal event (top right) and events are highlighted within an expanded 2 min sample trace (bottom). Compared to vehicle, 30 and 100 mg/kg SB2193 increased SWD incidence **(A)**. SB2193 dose-dependently increased SWD total duration **(C)** and 100 mg/kg SB2193 increased average duration **(E)**. No significant effect of treatment on SWD average frequency was observed **(G)**. Similarly, raw data analyses revealed an increase in SWD incidence at 30 mg/kg, total duration at 30 and 100 mg/kg **(D)** and average duration at 100 mg/kg **(F)**. Values are reported as mean ± SEM and significance is denoted as *p* = 0.05*, 0.01** or 0.001***. Males and females were pooled for these analyses; however, sex has been differentiated in the figures for clarity (circles, males; squares, females). *BL*: baseline; *Veh*: vehicle; *Avg*: average. See Results for details of statistical analyses.

Absence seizures are associated with T-type channel dysfunction in thalamocortical circuits, thus we tested the effect of SB2193 on SWD activity in male and female GAERS (Cain and Snutch, 2013). EEG activity was recorded for 4 h following SB2193 administration and SWDs were assessed by 1) incidence, 2) total duration, 3) average duration, and 4) frequency of seizures (Figure 8). Results are presented as normalized SWD data showing the percentage change in SWD activity compared to baseline sessions (Figures 8A, C, E, G), and raw SWD data as sampled over the 4 h recording window (Figures 8B, D, F, H). Main effects were assessed for treatment (10, 30, 100 mg/kg) and time (1, 2, 3, 4 h). Unexpectedly, SB2193 (30 mg/kg) increased SWD incidence and the two highest doses of SB2193 tested increased total duration and average duration of SWDs in GAERS.

For normalized SWD data, main effects of treatment were observed for SWD incidence [Figure 8A; F (3, 39) = 3.58; *p* ≤ 0.05], total duration [Figure 8C; F (3, 39) = 7.21; *p* ≤ 0.001], and average duration [Figure 8E; F (3, 39) = 4.73; *p* ≤ 0.01]. Dunnett’s *post hoc* analysis revealed SB2193 significantly increased SWD incidence and duration at 30 and 100 mg/kg doses and increased average SWD duration at 100 mg/kg compared to vehicle (all *p* ≤ 0.05). Finally, a main effect of time was observed for SWD incidence [Figure 8A; F (3, 39) = 4.39; *p* ≤ 0.01] and total duration [Figure 8C; F (3, 39) = 2.87; *p* ≤ 0.05]; *post hoc* analyses revealed that these increases occurred between 1 and 3 h (*p* ≤ 0.05). No significant effects of SB2193 on SWDs were observed in the first hour following administration (Supplementary Figure S2).

Analyses of raw count data showed a main effect of treatment for SWD incidence [Figure 8B; F (2.7, 35.18) = 6.46; *p* ≤ 0.05], total SWD duration [Figure 8D; F (2.6, 34.4) = 7.67; *p* ≤ 0.001], and average SWD duration [Figure 8F; F (1.9, 24.9) = 3.77; *p* ≤ 0.05]. Dunnett’s multiple comparisons test showed SB2193 significantly increased SWD incidence at 30 mg/kg dose compared to vehicle (*p* ≤ 0.05), SWD total duration at 30 (*p* ≤ 0.05) and 100 (*p* ≤ 0.001) mg/kg doses compared to vehicle, and SWD average duration at 100 mg/kg (*p* ≤ 0.05). No changes in SWD frequency were observed with treatment.

## 4 Discussion

Here we report the pharmacological characterisation of the MEPIRAPIM analogues SB2193 and SB2193F as potent, brain-penetrant inhibitors of Ca_v_3 channels. In mouse models of epilepsy, SB2193 provided acute protection against 6 Hz-induced seizures but did not affect spontaneous seizures in *Scn1a* ^*+/−*^ mice. Contrastingly, higher doses of SB2193 increased SWD incidence and duration in GAERS.

The potency of MEPIRAPIM analogues at T-type channel subtypes had not been previously established. Our data indicate that SB2193 and SB2193F are pan Ca_v_3 channel inhibitors, exhibiting sub-micromolar to low micromolar potencies at Ca_v_3.1, Ca_v_3.2, and Ca_v_3.3 channel subtypes (IC_50_ range = 0.75—8.6 μM). One aim of recent drug discovery efforts has been the development of subtype selective Ca_v_3 channel inhibitors. Unfortunately, the high level of homology in the exofacial structure of T-type channels has so far impeded the development of selective ligands (Weiss and Zamponi, 2019). Targeting the endofacial region of the channel represents a promising strategy for developing selective inhibitors (Snutch and Zamponi, 2018). The recent resolution of the cryo-EM structures of Ca_v_3.1 and Ca_v_3.3 may guide subsequent design of ligands which bind in this region (Zhao et al., 2019; He et al., 2022).

*In silico* docking to the cryo-EM structure of Ca_v_3.1 suggests that the MEPIRAPIM analogues SB2193 and SB2193F may bind in the channel pore region occupied by the antagonist Z944 in the cryo-EM structure. Similarly to Z944, the calculated poses for both SB2193 and SB2193F feature hydrogen bond interactions between a carbonyl oxygen and the Lys1462 residue. The overall calculated binding pose of both SB2193 and SB2193F is analogous to that of Z944, with the polar ester and piperazine regions occupying the same space as the peptide bonds and piperidine group of Z944, and coordinated by the same polar residues. The 6-membered aromatic ring of the indole in SB2193 and SB2193F occupies the same region as the phenyl ring of the Z944 ligand, positioned nearly perpendicular to the pore axis (Zhao et al., 2019).

The finding that SB2193 and SB2193F inhibited T-type channels is consistent with prior research showing that endocannabinoids, phytocannabinoids, and synthetic cannabinoids share this property (Chemin et al., 2001; Ross et al., 2008; Bladen et al., 2021; Mirlohi et al., 2022; Udoh et al., 2022). SB2193 and SB2193F are structural analogues of MEPIRAPIM and, like the parent compound, have minimal activity at cannabinoid CB_1_ receptors (Kevin et al., 2022). Thus, the MEPIRAPIM backbone might offer a viable new chemotype to develop T-type channel inhibitors lacking cannabimimetic activity. Notwithstanding this, it is conceivable that dual T-type and cannabinoid receptor activity may enhance therapeutic effects, particularly in pain and epilepsy models in which both are relevant targets (Roebuck et al., 2021; Anderson et al., 2022; Sugaya and Kano, 2022; Zieglgänsberger et al., 2022).

After establishing the *in vitro* potency of SB2193 and SB2193F, we then sought to characterise their *in vivo* pharmacokinetic properties and brain uptake. Pharmacokinetic analysis revealed both compounds to be brain penetrant, achieving low micromolar brain concentrations following a single 10 mg/kg i. p. injection in mice. SB2193 displayed particularly high brain penetrance, exhibiting a brain-plasma ratio of 2.7. Somewhat surprisingly, while the introduction of fluorine is commonly used as a strategy to improve brain uptake, here the addition of a fluorine in SB2193F did not improve brain penetrance (Sun and Adejare, 2006).

Based on the favourable potency of SB2193 at inhibiting T-type channels and its optimal brain uptake, the anticonvulsant activity of this compound was assessed in several established animal seizure models*.* The anticonvulsant activity of SB2193 was first assayed in the *Scn1a* ^*+/−*^ mouse model which recapitulates the characteristic symptoms of Dravet syndrome, including the development of spontaneous seizures and premature mortality (Miller et al., 2014). Ca_v_3.1 was previously identified as a genetic modifier of epilepsy in *Scn1a* ^*+/−*^ mice, as genetic knockdown of the Ca_v_3.1-encoding gene *Cacna1g* was reported to ameliorate spontaneous seizures and improve survival (Calhoun et al., 2017). We therefore reasoned that SB2193 may be protective in this model. Contrary to expectations, sub-chronic treatment with SB2193 had no effect on spontaneous seizure frequency or survival in *Scn1a* ^*+/−*^ mice. Although sub-chronic oral administration of SB2193 achieved low micromolar brain concentrations (1.7 ± 0.6 μM), these values fall above the Ca_v_3.1 IC_50_ using the FLIPR assay (0.75 µM) but below the IC_50_ (3.5 μM) as determined by whole cell patch-clamp electrophysiology. Therefore, we cannot exclude the possibility that low brain concentrations of SB2193 may have contributed to the absence of a protective effect in the *Scn1a* ^*+/−*^ mice. To provide more definitive data on whether T-type inhibition is useful in treating Dravet syndrome, a future study could assess the effects of selective T-type calcium channel inhibitors with improved oral pharmacokinetic properties in the *Scn1a* ^*+/−*^ mice.

However, this result may also suggest that pan T-type inhibition may be an ineffective treatment strategy for Dravet syndrome. Consistent with our findings here, the pan T-type inhibitor ethosuximide was ineffective against seizures in an *Scn1a* ^*+/−*^ zebrafish model (Baraban et al., 2013). As SB2193 inhibits not only Ca_v_3.1 but also Ca_v_3.2 and Ca_v_3.3, it is possible that inhibition of these additional T-type channel subtypes may negate any protective effect of Ca_v_3.1 inhibition in this model. Notably, Ca_v_3.3 null mice exhibit increased susceptibility to SWDs suggesting that Ca_v_3.3 inhibition may be proconvulsant (Lee et al., 2014). In this regard, selective inhibition of Ca_v_3.1 may be desirable for the development of novel anticonvulsants for the treatment of Dravet syndrome.

SB2193 was also screened in the 6 Hz mouse model of psychomotor seizures. This acute electrically-induced seizure model is utilised in the preclinical Epilepsy Screening Therapy Program (ETSP) at the University of Utah to identify anticonvulsant agents (Barker-Haliski et al., 2017; Wilcox et al., 2020). A single 100 mg/kg dose of SB2193 significantly increased the percentage of mice protected from 6 Hz seizures. The ED_50_ of SB2193 was approximately 120 mg/kg, indicating greater potency than ethosuximide in this model (ED_50_ > 600 mg/kg) (Barton et al., 2001). This result is also consistent with SB2193 displaying more potent Ca_v_3 channel inhibition than ethosuximide, with the latter displaying potency in the millimolar concentration range (Todorovic et al., 2000). Unfortunately, poor aqueous solubility prevented assessment of the effect of SB2193 at doses above 100 mg/kg.

The 6 Hz seizure model produces intense activation of temporal lobe structures, including the dentate gyrus which is implicated in the neuropathology of temporal lobe epilepsy (Barton et al., 2001; Scharfman, 2019). The efficacy of SB2193 against 6 Hz induced seizures therefore raises the possibility that this compound may also be protective in temporal lobe epilepsy. This hypothesis is supported by the finding that the pan T-type channel blocker, Z944, protected against seizures in the amygdala kindling model and kainic acid induced *status epilepticus* rat models of temporal lobe epilepsy (Casillas-Espinosa et al., 2015; Casillas-Espinosa et al., 2019). Future studies might examine whether SB2193 has anticonvulsant effects in other animal models of temporal lobe epilepsy, including the kainic acid and pilocarpine models (Cavalheiro et al., 1996).

Finally, SB2193 was screened for anticonvulsant activity in GAERS. These animals display reliable spike and wave discharges (SWDs) characteristic of childhood absence epilepsy (CAE) (Tringham et al., 2012; Marks et al., 2019; Roebuck et al., 2021; McElroy et al., 2022; Roebuck et al., 2022). The GAERS line possesses a gain-of-function mutation in Ca_v_3.2 which is believed to contribute to absence seizures (Tringham et al., 2012; Proft et al., 2017; Cain et al., 2018). Given that the T-type inhibitors ethosuximide and Z944 both reduce absence seizures in these animals, we hypothesised that SB2193 might also be protective in this model (Tringham et al., 2012; Dezsi et al., 2013). Surprisingly, SB2193 treatment increased SWD incidence and duration in GAERS over a 4-h recording period. Given the close exofacial sequence homology of Ca_v_3 channels with other voltage-gated ion channels, it is possible that this effect was due to off-target actions of SB2193 at other targets such as voltage-gated sodium channels. Sodium channel blockers such as phenytoin and carbamazepine are known to aggravate seizures in GAERS (Gurbanova et al., 2006; Liu et al., 2006). To address this potential liability, MEPIRAPIM analogues should be screened for activity at sodium channels in future work.

It should also be noted that the diversity of human epilepsies and various animal models represent a highly complex set of underlying molecular, cellular, and circuit pathophysiologies. The anti-seizure efficacy of an agent against one type of epilepsy or animal model may not necessarily extend to other epilepsies and models, and in some cases may worsen the seizure phenotype. Together with the distinct regional expression patterns and roles that the three T-type channel subtypes contribute toward seizure activity, it is unlikely that single target therapeutics will effectively treat multiple types of epilepsy (Cain and Snutch, 2013; Cain et al., 2018). Slight differences in brain exposure levels of SB2193 across animal models may have also contributed to the divergent effects observed in the present study. Additionally, the free fraction of SB2193 was not determined in our study, which would help provide a more definitive evaluation of target engagement. Future studies investigating the plasma protein binding of this compound may clarify exposure of the active drug *in vivo.* Moreover, our predictions of potency here were determined against human channels, and it is possible that the potency of these compounds against mouse and rat variants of the Ca_v_3 channels may differ.

Finally, given the structural similarities between SB2193 and potent SCRAs, we speculate that this analogue could undergo bioconversion to a CB_1_ receptor agonist active metabolite *in vivo*. This conjecture is strengthened by the finding that the proconvulsant effect of SB2193 in GAERS animals was delayed beyond the period of peak brain concentrations of the parent drug observed in mice. CB_1_ receptor agonism may be proconvulsant, as the CB_1_ partial agonist Δ^9^-tetrahydrocannabinol (THC) increased SWD frequency in GAERS (Roebuck et al., 2022). The Boc group of SB2193 is a likely site of metabolism, as these groups are readily cleaved (Han et al., 2001). Indeed, earlier structure-activity data showed that removal of the Boc group from the SB2193 scaffold is sufficient to partially restore functional activity at CB_1_ receptors (Kevin et al., 2022). This possibility could be investigated in future studies by determining the metabolites of SB2193 and assessing whether they have activity at cannabinoid receptors.

In conclusion, the MEPIRAPIM analogue SB2193 has acute anticonvulsant effects in the 6 Hz mouse model of psychomotor seizures but not the *Scn1a* ^*+/−*^ mouse model of Dravet syndrome, nor the GAERS model of absence epilepsy. These results provide proof of principle that brain-penetrant MEPIRAPIM analogues provide novel chemical scaffolds to advance T-type inhibitors against psychomotor seizures. However, these data also highlight some potential liabilities of this chemotype in certain types of epilepsy.

## Data Availability

The raw data supporting the conclusion of this article will be made available by the authors, without undue reservation.

## Supplementary material

SUPPLEMENTARY FIGURE S1 Percentage weight gain of *Scn1a* ^*+/−*^ mice sub-chronically administered SB2193-treated and untreated chow. Chow supplementation began on P18 and continued to P30 with mouse bodyweights recorded on P25 and P31. Data represent percentage change in bodyweight from P18 (mean ± SEM, *n* = 14). REML mixed-effects analysis indicated a significant main effect of time [F (1, 4) = 39.12, *p* = 0.003] but no effect of treatment [F (1, 8) = 0.54, *p* = 0.5] and treatment x time [F (1, 4) = 0.084, *p* = 0.8] on bodyweight change.

SUPPLEMENTARY FIGURE 2 Effects of SB2193 in GAERS during first 60 min post-administration. SWDs were assessed in 10 min time-bins during the first hour of EEG recordings beginning ∼20 min after SB2193 treatment (i.p., Veh, 10, 30, 100 mg/kg). Top panels show SWD incidence , **(A)** total duration **(C)**, average duration **(E)**, and average frequency **(G)**. Data has been normalized to percentage change from baseline and compared to vehicle treatments. Bottom panels show raw SWD incidence **(B)**, total duration **(D)**, average duration **(F)** and average frequency **(H)** in the first 1 h of recordings. Statistical analyses revealed no significant differences between treatments. Values are reported as mean ± SEM. Males and females were pooled for these analyses; however, sex has been differentiated in the figures for clarity. *BL*: baseline; *Veh*: vehicle; *Avg*: average.

SUPPLEMENTARY TABLE S1 Liquid chromatography-tandem mass spectrometry analyte mass transitions and collision energies.
