# Supplementary material for: MEPIRAPIM-derived synthetic cannabinoids inhibit T-type calcium channels with divergent effects on seizures in rodent models of epilepsy
Source: Front Physiol. 2023 Apr 4;14:1086243. doi: 10.3389/fphys.2023.1086243 (PMC10110893; doi:10.3389/fphys.2023.1086243)
Supplement: Supplementary file 1 [file Table1.docx]

| **Analyte** | **Molecular weight** | **Precursor and product ions (m/z)** | |
| --- | --- | --- | --- |
| SB2193 | 399.54 | 400.2 > 344.25 |  |
|  |  | 400.2 > 214.25 |  |
|  |  | 400.2 > 144.1 |  |
| SB2193F | 417.53 | 418.2 > 362.2 |  |
|  |  | 418.2 > 232.15 |  |
|  |  | 418.2 > 144.2 |  |
| Diazepam | 284.7 | 284.6 > 257 |  |
|  |  | - 1. > 220 |  |

**SUPPLEMENTARY TABLE S1**

Liquid chromatography-tandem mass spectrometry analyte mass transitions

and collision energies.
