# Supplementary figures and images for: MEPIRAPIM-derived synthetic cannabinoids inhibit T-type calcium channels with divergent effects on seizures in rodent models of epilepsy
Source: Front Physiol. 2023 Apr 4;14:1086243. doi: 10.3389/fphys.2023.1086243 (PMC10110893; doi:10.3389/fphys.2023.1086243)

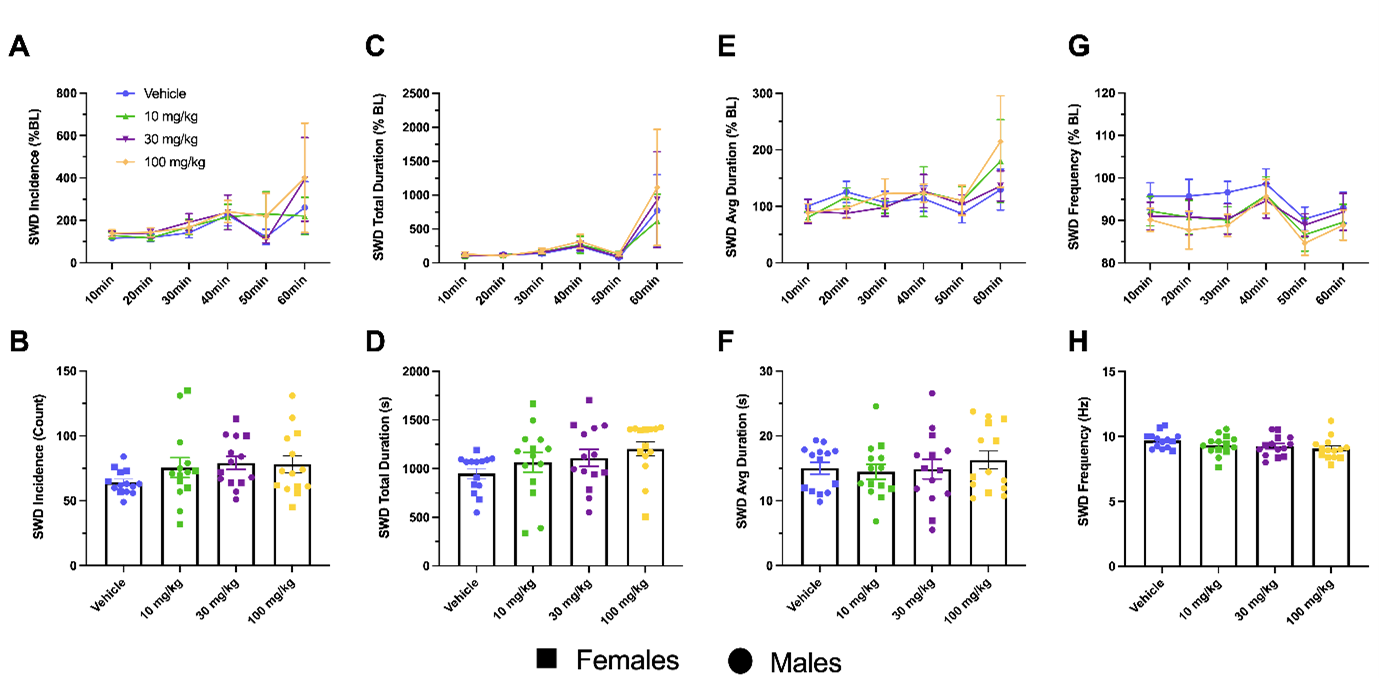

Supplement: Supplementary file 2 [file Image2.TIF]

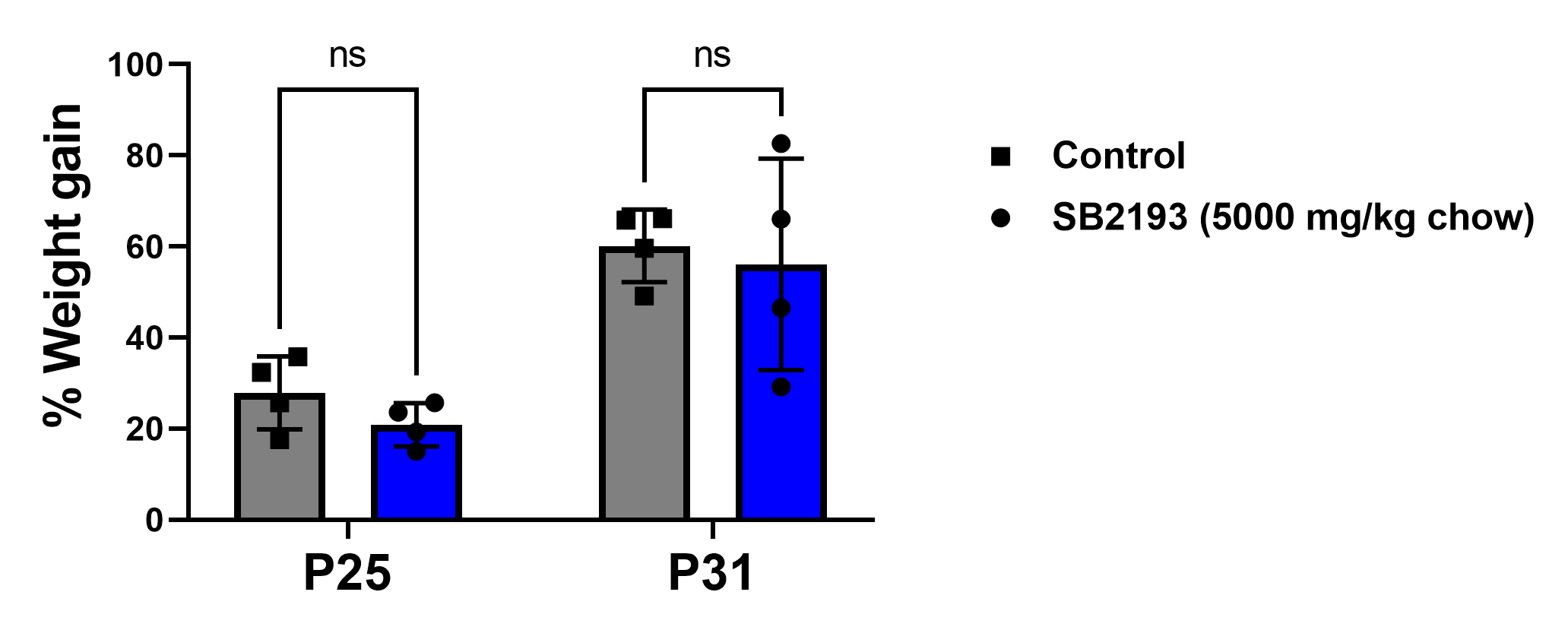

Supplement: Supplementary file 3 [file Image1.TIF]
